# Analysis of degradation products of Novichok agents in human urine by hydrophilic interaction liquid chromatography–tandem mass spectrometry

**DOI:** 10.1007/s11419-022-00656-4

**Published:** 2022-12-31

**Authors:** Mai Otsuka, Akinori Yamaguchi, Hajime Miyaguchi

**Affiliations:** National Research Institute of Police Science, 6-3-1 Kashiwanoha, Kashiwa, Chiba 277-0882 Japan

**Keywords:** Warfare agents, HILIC–MS/MS, Nerve agents, Novichok agents, Biological fluids

## Abstract

**Purpose:**

The detection of hydrolysis products of Novichok agents in biological samples from victims is important for confirming exposure to these agents. However, Novichok agents are new class of nerve agent and there have been only few reports on analyses of Novichok agent degradation products. Here, we developed hydrophilic interaction liquid chromatography (HILIC)–tandem mass spectrometry (MS/MS) methods to detect Novichok agent degradation products in human urine with simple pretreatment and high sensitivity.

**Methods:**

A Poroshell 120 HILIC-Z column was used to analyze six Novichok agent degradation products. For urine samples, we used a simple pretreatment method, which consisted of deproteinization with acetonitrile and microfiltration. We calculated the p*K*_a_ values of the OH groups, the log P values, and the molecular weights to investigate the difference in chromatographic behaviors of the Novichok agent degradation products and the degradation products of conventional nerve agents.

**Results:**

Six Novichok agent degradation products, including *N*-(bis-(diethylamino)methylidene)-methylphosphonamidic acid (MPGA), which could not be detected by our previous method, could be analyzed with sufficient peak shape and mutual separation. The detection limits of six Novichok agent degradation products were sufficiently low (1–50 ng/mL) and the calibration curves showed sufficient linearity. The physicochemical parameters of Novichok agent degradation products were different from those of conventional nerve agent degradation products, and this explains the difference in chromatographic behaviors.

**Conclusion:**

Six Novichok agent degradation products were successfully analyzed by HILIC–MS/MS. Due to the absence of a derivatization step, throughput performance was higher than our previous derivatization-liquid chromatography–MS/MS method.

**Supplementary Information:**

The online version contains supplementary material available at 10.1007/s11419-022-00656-4.

## Introduction

**Fig. 1:**
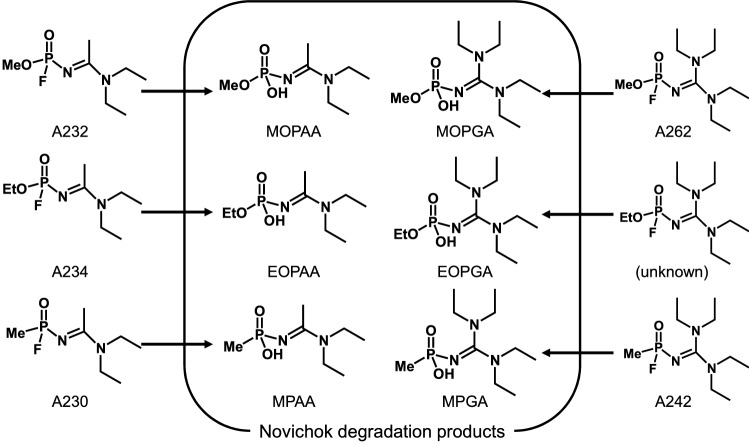
Hydrolysis pathways of Novichok agents

Nerve agents, such as sarin and VX, are highly toxic organophosphorus compounds and they strongly inhibit the activity of cholinesterase [1]. They are classified as chemical warfare agents, and their production, storage, and use are strictly controlled internationally [2]. Nevertheless, these compounds have been used in various crimes, such as terrorist attacks and murders [3–5]. In addition to conventional nerve agents, a new class of nerve agents called Novichok agents have also been used recently. In 2018, two intoxication cases with Novichok agents occurred in the United Kingdom [6]. More recently, a Novichok agent was used in an airplane in 2020 [7]. Novichok agents were added to the controlled chemicals list in the Chemical Weapons Convention in November 2019. Although Novichok agents have been investigated experimentally or with theoretical calculations [8–12], little is known about their toxicity, synthetic methods, and analytical methods. When Novichok agents are used in crimes, verification of the exposure to the agents by analyzing biological samples from victims is important. However, the detection of intact agents is difficult because of their high reactivity. Thus, detection of the hydrolysis products or protein adducts of Novichok agents is important, similar to other nerve agents [13, 14]. For the verification of Novichok agents exposure, the analysis of human butyrylcholinesterase adducts of Novichoks A230, A232, and A234 has been reported [12, 15], but this method can only be applied to blood samples. Before we started our study, there was only one analysis of Novichok degradation products [16], although only the degradation products of A234 were analyzed, and there was no information about limits of detection (LODs) or quantitative results. Previously, we calculated the hydrolysis pathway of Novichok agents and compared the results with conventional nerve agents [17]. We found that Novichok agents behaved more like VX than sarin, even though Novichok agents have an F atom as a leaving group, similar to sarin. In the Novichok agents, F^−^ was released more easily than the alkyl amine. A plausible hydrolysis pathway for Novichok agents is shown in Fig. 1. We have developed an analytical method for these degradation products in human urine by dimethoxytriadinylation liquid chromatography (LC)–tandem mass spectrometry (MS/MS) [18]. This was the first report of a screening method for Novichok agent degradation products, and the LODs (determined as the concentration at which each selected product ion intensity was more than 100 ion counts) were sufficiently low for most compounds. However, there were some limitations to the method. First, because the method involved derivatization (50 ℃ for 2 h), the procedure needed long operation times as a screening method. Second, the linearity of the calibration curves decreased for some compounds (*R*^2^: 0.936–0.988 for five Novichok agent degradation products), which may be due to the variation in the derivatization reaction. Third, most importantly, the degradation product of A242, *N*-(bis-(diethylamino)methylidene)-methylphosphonamidic acid (MPGA), could not be detected at all due to the low reactivity of MPGA. Thus, we decided to investigate a direct analytical method using LC–MS/MS with hydrophilic interaction liquid chromatography (HILIC) columns to analyze Novichok agent hydrolysis products in human urine with a simple pretreatment, high stability, and high sensitivity.

## Materials and methods

### Materials

The Novichok A-series degradation products, *O*-methyl (1-(diethylamino)-ethylidene)phosphoramidic acid (MOPAA), *O*-ethyl (1-(diethylamino)ethylidene)phosphoramidic acid (EOPAA), *N*-(1-(diethylamino)ethylidene)-methylphosphonamidic acid (MPAA), *O*-methyl (bis(diethylamino)methylidene)phosphoramidic acid (MOPGA), *O*-ethyl (bis(diethylamino)methylidene)phosphoramidic acid (EOPGA), and MPGA, were synthesized by newly developed methods in our laboratory [18]. All aqueous solutions were produced using LC–MS-grade water (FUJIFILM Wako Pure Chemical Co., Osaka, Japan). Drug-free urine from five individual donors (three males and two females) was purchased from Lee Biosolutions, Inc. (Maryland Heights, MO) and stored at − 20 °C until use. All other chemicals were used as received. The urine samples were spiked with Novichok agent degradation products and left at room temperature for 1 h or longer before the analyses.

### Hydrophilic interaction liquid chromatography–MS/MS conditions

The HILIC–MS/MS analyses were performed using a liquid chromatograph (Ultimate3000, Thermo Fisher Scientific, Waltham, MA) connected to a mass spectrometer (TSQ Fortis, Thermo Fisher Scientific). A Poroshell 120 HILIC-Z column (Agilent Technologies, Santa Clara, CA; 150 mm × 2.1 mm I.D., 2.7 μm particle size) was used as a stationary phase. The column temperature was maintained at 40 ℃ and the flow rate was 0.20 mL/min. Solvent A (15 mM NH_4_OAc buffer, pH 4.8) and solvent B (acetonitrile (MeCN)) were used in gradient mode with the following program: 90% B for 2 min, decreased to 75% B at 1%/min, decreased to 40% B at 7%/min, 40% B for 5 min, increased to 90% B at 10% B/min, and 90% B for 10 min. The injection volume was 1 μL. The mass spectrometer was used in electrospray ionization (ESI) positive mode and the ionization voltage was 3.5 kV. The ion source temperature was 300 ℃ and the collision gas was argon. The optimized multiple reaction monitoring (MRM) parameters are summarized in Table 1.

**Table 1:** Optimized MRM parameters

Target compounds	Precursor ion (*m/z*)	Product ion(*m/z*)	Collision energy (V)	Minimum dwell time (ms)	Tube lens (V)
MOPAA	209.088	74.196	14.31	65.002	52
		136.042	14.86	65.002	52
EOPAA	223.125	74.125	14.94	65.002	62
		150.042	13.68	65.002	62
MPAA	193.000	74.000	15.00	65.002	50
		120.000	15.00	65.002	50
MOPGA	266.000	99.000	22.00	65.002	68
		193.000	16.00	65.002	68
EOPGA	280.225	99.083	23.87	65.002	78
		207.125	15.83	65.002	78
MPGA	250.312	73.982	17.72	65.002	65
		99.226	21.39	65.002	65

### Detection limits

The LODs were determined based on the guidelines of the Organisation for the Prohibition of Chemical Weapons Biomedical Proficiency Test [19, 20]. The tolerance for the ratio of peak areas for the two product ions was set. The detection was determined when the obtained peak area ratio of the sample was within the tolerance in all the attempted analyses at the same concentration (*n* = 3). The relative intensities of the two ions and tolerances for each compound are shown in Table S1.

### Pretreatment of urine

MeCN (200 μL) was added to the urine samples (50 μL) and vortexed for 30 s. The samples were centrifuged at 12,000 × *g* for 5 min, and then the supernatants were filtered by Cosmospin Filter G (0.2 μm, Nacalai Tesque, Kyoto, Japan). The filtrates were moved into LC vials and analyzed by HILIC–MS/MS. We also investigated ultrafiltration (Amicon Ultra-0.5, 3 kDa, Merck Millipore, Burlington, MA) instead of microfiltration.

### Matrix effects

For the pretreatment described above, the matrix effects were examined. The same amount of MeCN was added to water or blank urine (50 μL), and the sample was vortexed and centrifuged at 12,000 × *g* for 5 min. The supernatants were filtered by Cosmospin Filter G, and then the same amount of standard solution of Novichok agent degradation products was added to the filtrates and analyzed by HILIC–MS/MS. The matrix effect values were expressed as the ratio of the peak area of the urine sample to that of the water sample (set as 100%).

### Accuracy of the methods

The accuracies of the methods were determined by analyzing samples containing known concentrations of Novichok agent degradation products using calibration curves constructed using between five and eight points with a 1/*x* weighting factor, and three samples were prepared for each concentration. The samples containing known Novichok agent degradation product concentrations were analyzed three times.

### Calculation of physicochemical parameters

Molecular weights were calculated by ChemDraw Standard (CambridgeSoft, Cambridge, UK), log P was calculated with ALOGPS 2.1 [21, 22], and p*K*_a_ was calculated with Gaussian 09 [23] or Gaussian 16 [24] according to the literature [25]. After the geometry optimization of the molecule and its ionized form at the Becke’s three-parameter exchange function and the Lee–Yang–Parr nonlocal correlation functional (B3LYP)/6–31 + G** level [26–29], single-point energy calculations were done at the M062X/6–311 +  + G** level [30]. The SMD free energy of solvation (H_2_O) was calculated at the M062X/6–311 +  + G** level. The p*K*_a_ value noted in Evans’s p*K*_a_ table [31] of acetic acid was used as the reference compound and p*K*_a_ values of target compounds were obtained according to Equation S1. The number of imaginary frequencies was 0 for the minima.

## Results

### Optimization of HILIC–MS/MS conditions

**Fig. 2:**
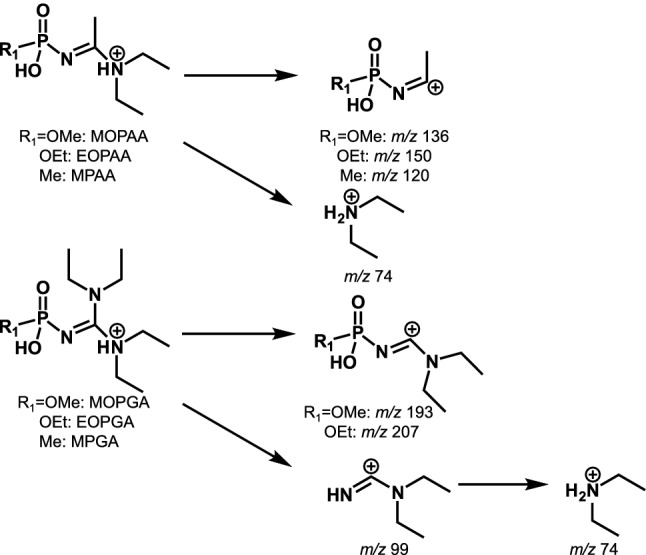
Predicted MS/MS fragmentation pathways

**Fig. 3:**
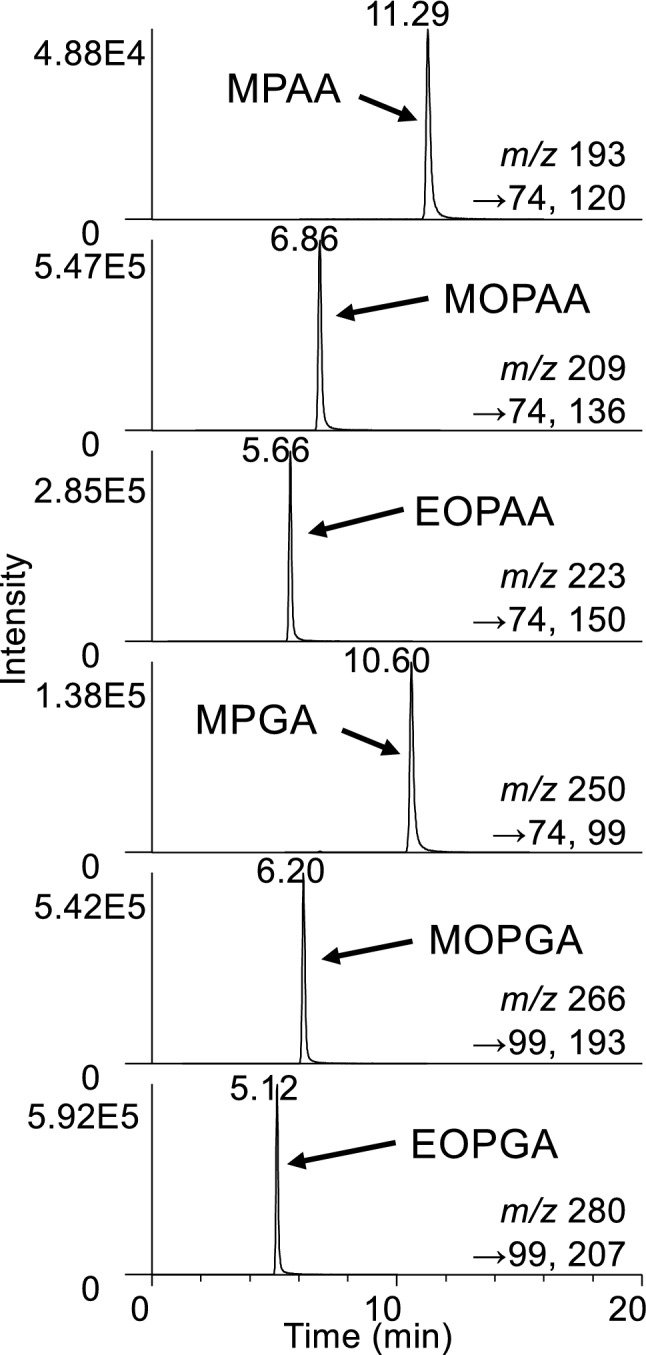
EIC obtained using the HILIC column

First, we optimized the MS/MS parameters automatically for each Novichok agent degradation product using the option in the TSQ Fortis mass spectrometer. The analysis with positive-mode ESI showed larger peak intensities than that with negative-mode ESI. The reason for this will be discussed later in this article. The optimized parameters are described in the Materials and methods section (Table 1). For MPAA and MOPGA, the parameters were optimized manually. The predicted fragmentation pathways are shown in Fig. 2. Next, we investigated the LC conditions. Initially, we investigated the conditions of ion chromatography (IC)–MS/MS, which were effective for analyzing the degradation products of conventional nerve agents [32], but we did not obtain satisfactory peak shapes by IC–MS/MS with the anion-exchange column (data not shown). This may have been due to the zwitterionic character of the Novichok agent degradation products, which have both OH groups and amidine or guanidine groups. We also investigated a widely used reversed-phase separation column, but large base line variations were observed, and reproducible peak areas were not obtained (data not shown). Then we decided to investigate the Agilent HILIC-Z column, which has a sulfobetaine zwitterionic surface [33]. We have used this column for the analysis of nitrogen mustard degradation products, and the column showed sharp peak shapes and stable retention times compared with a silica-based HILIC column modified with phosphorylcholine [34]. The optimized extracted ion chromatogram for the analysis of Novichok agent degradation products with the Agilent HILIC-Z column is shown in Fig. 3. The six Novichok agent degradation products were separated well and the peak shapes were sharp. Next, the variation in the retention times of Novichok agent degradation products was investigated (Table S2). In general, HILIC columns need a longer equilibration time than reversed-phase columns, and large retention time variations are observed when the equilibration time after the gradient is not sufficient [35]. However, the retention times showed small variations within a day or within a week in this study. Although large variations were observed over a long period (4 months), the retention times were stable enough for practical use. The optimal HILIC–MS/MS conditions are summarized in the Experimental section.

### Analysis of urine samples

We developed a method to analyze Novichok agent degradation products in urine samples. First, we examined a procedure that consisted of dilution with water (dilution ratios of 20, 10, and 5) and deproteinization with ultrafiltration by Amicon Ultra-0.5. However, the peak shapes were poor and peak cracking was observed for some compounds (Fig. S1 (a)). Then we examined a procedure that consisted of deproteinization with MeCN and microfiltration (Cosmospin Filter G), and the corresponding chromatogram is shown in Fig. S1 (b). The peak shapes were satisfactory for all six Novichok agent degradation products. Under the optimized conditions, the LODs were determined for urine samples. The LODs were 50, 5, 5, 1, 1, and 7.5 ng/mL for MOPAA, EOPAA, MPAA, MOPGA, EOPGA, and MPGA, respectively. For MOPAA, interfering peaks were observed in the extracted ion chromatograms (EICs, *m/z* 209 → 136) and the peaks increased the LOD of MOPAA, even though the MOPAA peak could be seen clearly in the EIC of another MS/MS transition (*m/z* 209 → 74, Fig. S2).

Next, the linearity of the calibration curves was investigated. The equations, coefficient of determination (*R*^2^), and linear ranges for urine samples are summarized in Table 2. The calibration curves showed sufficient linearity over a wide concentration range. In addition, the *R*^2^ values in this study (0.976–0.989, Table 2) were superior to those in the previous study [18] (0.936, 0.988, 0.981, 0.936, and 0.963 for MOPAA, EOPAA, MPAA, MOPGA, and EOPGA, respectively). This might be because we analyzed the degradation compounds directly and the variation in sample pretreatment such as variation of reaction efficiencies was reduced. The relative errors of each point in the calibration curves are summarized in Table S3. In the linear ranges examined, no outliers were observed and the lowest concentrations of the calibration curves were taken as the limits of quantitation.

**Table 2:** Calibration curves for urine samples

Target compound	Equation	*R*^2^	Linear range (ng/mL)
MOPAA	$y= 4570.0 x + 35913$	0.976	50–1000
EOPAA	$y= 11167 x + 1466.2$	0.982	5.0–1000
MPAA	$y= 770.72 x + 1101.1$	0.988	5.0–1000
MOPGA	$y= 2870.4 x + 1289.8$	0.985	1.0–1000
EOPGA	$y= 2156.0 x + 244.80$	0.984	1.0–1000
MPGA	$y= 1231.9 x + 2988.2$	0.989	7.5–1000

### Validation of the methods

To validate the methods, we conducted blank tests, matrix effects tests, and accuracy tests for urine samples. For the blank tests, blank urine samples obtained from five donors were analyzed as described above, and no peaks with similar retention times to the Novichok agent degradation products were detected (data not shown). Next, we investigated the matrix effects of the urine samples (Table S4). Large ion enhancement was observed for MOPAA, MPAA, and MPGA, instead large ion suppression was observed for EOPAA and MOPGA. Thus, the matrix of the samples for calibration curves should be urine rather than solvents to obtain precise quantitation results. Next, accuracy tests were performed (Table 3). The concentrations of Novichok agent degradation products in urine samples were estimated sufficiently when the calibration curve samples and the samples for evaluation were analyzed on the same day (intra-day). When we used the calibration curves to estimate the concentration of the samples measured on different days (inter-day), the quantitation results were also sufficient in most cases, except for MPAA. The reason of large inter-day variation for MPAA is unclear, but one reason might be the low ionization efficiency of MPAA and the consequent small slope of the calibration curve. When the slope of calibration curve would be small, the quantitation results would be highly affected by variation of peak areas. Thus, unknown samples and calibration curve samples can be analyzed on different days for the analysis of most Novichok agent degradation products, but they should be analyzed on the same day to obtain accurate quantitation results for MPAA.

**Table 3:** Accuracy tests for urine samples (%, average ± standard deviation, *n* = 3)

	Concentration (ng/mL)	MOPAA	EOPAA	MPAA	MOPGA	EOPGA	MPGA
Intra-day^a^	750	80 ± 5.3	77 ± 8.0	76 ± 4.0	79 ± 11	80 ± 14	77 ± 8.5
	75	106 ± 37	99 ± 43	98 ± 38	93 ± 40	91 ± 42	92 ± 39
	7.5	–	118 ± 5.4	117 ± 11	120 ± 4.2	126 ± 5.9	–
Inter-day^b^	750	70 ± 13	80 ± 13	109 ± 31	79 ± 15	88 ± 14	99 ± 21
	75	87 ± 42	107 ± 35	149 ± 8.5	103 ± 32	108 ± 27	126 ± 1.0
	7.5	–	94 ± 27	126 ± 5.7	97 ± 25	102 ± 19	–

^a^Three samples with known Novichok agent degradation product concentrations and samples for calibration curves (Table 2) were measured on the same day

^b^Three samples with known Novichok agent degradation product concentrations were measured on 3 days and the Novichok agent degradation product concentrations were estimated using the calibration curves constructed on 1 day (Table 2)

### Calculation of physicochemical parameters

To investigate the chromatographic behavior of Novichok agent degradation products with the HILIC column and compare it with the chromatographic behavior of the degradation products of conventional nerve agents, we calculated p*K*_a_ values of the OH groups, the log P values, and molecular weights of those compounds. Table 4 summarizes the results for the six Novichok agent degradation products and six conventional nerve agent degradation products (alkyl methyl phosphonic acids (RMPAs): methylphosphonic acid (MPA), a common product of nerve agent degradation; ethyl methylphosphonic acid (EMPA), a degradation product of VX; isopropyl methylphosphonic acid (IMPA), a degradation product of sarin; isobutyl methylphosphonic acid (^*i*^BuMPA), a degradation product of RVX; cyclohexyl methylphosphonic acid (CHMPA), a degradation product of cyclohexyl sarin; and pinacolyl methylphosphonic acid (PMPA), a degradation product of soman).

**Table 4:** Calculated physicochemical parameters

	p*K*_a_ of OH group	log P	Molecular weight
MOPAA	6.45	0.34	208.20
EOPAA	7.44	0.94	222.22
MPAA	7.34	0.39	192.20
MOPGA	8.80	0.98	265.29
EOPGA	7.97	2.22	279.32
MPGA	7.08	1.31	249.29
MPA	1.54	–0.89	96.02
EMPA	1.93	–0.32	124.08
IMPA	3.89	0.02	138.10
^*i*^BuMPA	2.04	0.24	152.13
CHMPA	2.42	0.85	178.17
PMPA	3.49	0.92	180.18

## Discussion

### Comparison of the present method with our previous study

We have recently reported an analytical method for Novichok agent degradation products involving derivatization [18]. Thus, we compare the present method with our previous method here [18]. Most importantly, our new method could detect MPGA, which was not detected by the previous method involving derivatization. For analytes that have relatively large steric hindrance for derivatization reactions, direct analysis rather than analysis involving derivatization is likely to give better analytical results.

For the chromatographic separation, the retention times of derivatized MOPAA and MPAA were similar and they could not be separated at the baseline. However, the six Novichok agent degradation products were separated sufficiently by the present method (Fig. 3). Although mutual separation may not be a problem when a mass spectrometer is used for detection, sufficient separation can reduce matrix effects for analytes in a complex matrix.

Next, we compared the LODs in the two methods. However, it should be noted that the LODs from the two methods cannot be compared strictly due to the difference in the type of mass spectrometer (triple quadrupole in the present study and Q-TOF in the previous study) and the way of determining LODs (using the peak area ratio in this study and using the signal intensity at which the selected product ion was ~ 100 ion counts in the previous study). The LOD of MOPAA was much higher in this study than in the previous study due to the interfering peak. For EOPAA and MPAA, the LODs were slightly lower in our previous study than in this study. In contrast, for MOPGA and EOPGA, the LODs were lower in this study than in the previous study, even though we used a more strict standard to determine LODs in this study. Moreover, MPGA, which could not be detected in the previous study, could be analyzed at a low LOD. Considering these results, the present method is the first convenient method for analyzing the six Novichok agent degradation products simultaneously with sufficient sensitivity.

### Investigation of the physicochemical parameters of Novichok agent degradation products

We calculated the physicochemical parameters of the Novichok agent degradation products and conventional nerve agent degradation products (RMPAs) to investigate the differences among the agents. For direct analysis with LC–ESI–MS, RMPAs have been analyzed with negative-mode ESI [36], whereas in this study, Novichok agent degradation products were analyzed with positive-mode ESI with high sensitivity. This difference may be due to the difference of acidity of OH group of the degradation compounds. The p*K*_a_ values of RMPAs were lower than that of acetic acid (p*K*_a_ 4.76), whereas Novichok agent degradation products showed high p*K*_a_ values (Table 4). Thus, Novichok agent degradation products may not be deprotonated easily under the neutral or weakly acidic conditions commonly used in LC–MS. In addition, Novichok agent degradation products have nitrogen atoms, which may show affinity for H^+^. Considering these results, it is reasonable for RMPAs to be analyzed with negative-mode ESI and for Novichok agent degradation products to be analyzed with positive-mode ESI with high sensitivity.

In our previous study, RMPAs were analyzed sufficiently with IC–MS/MS [32], whereas Novichok agent degradation products did not show satisfactory peak shapes. Moreover, although Novichok agent degradation products could be analyzed with HILIC–MS/MS as described above, RMPAs did not show sufficient peak shapes with the same HILIC column (data not shown). This might be due to the difference in hydrophilicities of those compounds. RMPAs, especially MPA, EMPA, and IMPA, showed low log P values, which correspond to high hydrophilicity (Table 4), whereas Novichok agent degradation products showed higher log P values. Although it is not the case for compounds with large hydrophilic moieties, the log P values are related to the size of molecules, which is related to molecular weights. For compounds in Table 4, Novichok agent degradation products have larger molecular weights than RMPAs. When compounds with similar molecular structures are considered, molecular weights might be able to be used to compare hydrophilicities. Considering the results above, although the high hydrophilicities of RMPAs were suitable for IC separations, the hydrophilicity of the Novichok agent degradation products might be too low for IC separations. We suggest that Novichok agent degradation products and RMPAs should be analyzed separately for high-sensitivity analysis. We are conducting further research to analyze these compounds simultaneously with high sensitivity.

## Conclusion

We developed an analytical method for Novichok agent degradation products using HILIC–MS/MS. Using this method, six Novichok agent degradation products were analyzed simultaneously, indicating that the method is suitable for efficient screening of Novichok agents’ exposure. The Novichok agent degradation products selected in this study are not inherent in human body, and detection of those compounds from urine samples provides a strong evidence of Novichok agents’ exposure. This study is the first reported analysis of MPGA, which is a degradation product of A242. Compared with previously reported derivatization-LC–MS/MS methods using reversed-phase columns, the mutual separations of the degradation products were improved. For urine samples, only a simple pretreatment, which consisted of deproteinization with MeCN and microfiltration, was needed to obtain sufficient chromatograms. The pretreatment took about 5 min and could be performed in parallel on many samples simultaneously. For HILIC–MS/MS analysis, about 42 min was needed per sample, and 34 samples could be tested per day. The absence of a derivatization step increased throughput compared with the derivatization-LC–MS/MS method, and our new method could be used for immediate verification of exposure, even after a large terrorist attack involving many casualties.

## Supplementary Information

Below is the link to the electronic supplementary material.

Supplementary file1 (pdf 438 KB)
