# Supplementary material for: Analysis of degradation products of Novichok agents in human urine by hydrophilic interaction liquid chromatography–tandem mass spectrometry
Source: Forensic Toxicol. 2022 Dec 31;41(2):221–9. doi: 10.1007/s11419-022-00656-4 (PMC10310577; doi:10.1007/s11419-022-00656-4)
Supplement: Supplementary file 1 — Supplementary file1 (pdf 438 KB) [file 11419_2022_656_MOESM1_ESM.pdf]

Supporting Information

1. Investigation of the HILIC-MS/MS conditions

1-1. HILIC-MS/MS analysis of standard samples of Novichok degradation products

Table S1. Selected fragment ions and tolerance of the ratio of those fragment ions

	MOPAA	EOPAA	MPAA	MOPGA	EOPGA	MPGA
Fragment ion 1 (*m/z*)	136	150	120	99	99	99
Fragment ion 2 (*m/z*)	74	74	74	193	207	74
Relative intensity (ion 1/ion 2)	77%	84%	55%	59%	67%	100%
Tolerance	±20%	±20%	±20%	±20%	±20%	±20%

1-2. Retention time variation

Table S2. Intra- and inter-day variation of retention times (min, n = 6)

	MOPAA	EOPAA	MPAA	MOPGA	EOPGA	MPGA
Intra-day	6.72 ± 0.01	5.52 ± 0.01	11.05 ± 0.01	6.04 ± 0.01	4.97 ± 0.01	10.32 ± 0.01
Within a week	6.66 ± 0.04	5.48 ± 0.04	10.99 ± 0.05	5.99 ± 0.04	4.92 ± 0.04	10.25 ± 0.06
Within 4 months	6.88 ± 0.15	5.65 ± 0.12	11.32 ± 0.27	6.22 ± 0.16	5.11 ± 0.13	10.64 ± 0.30

2. Investigation of the pretreatment methods of biological samples

2-1. Optimization of the pretreatment method

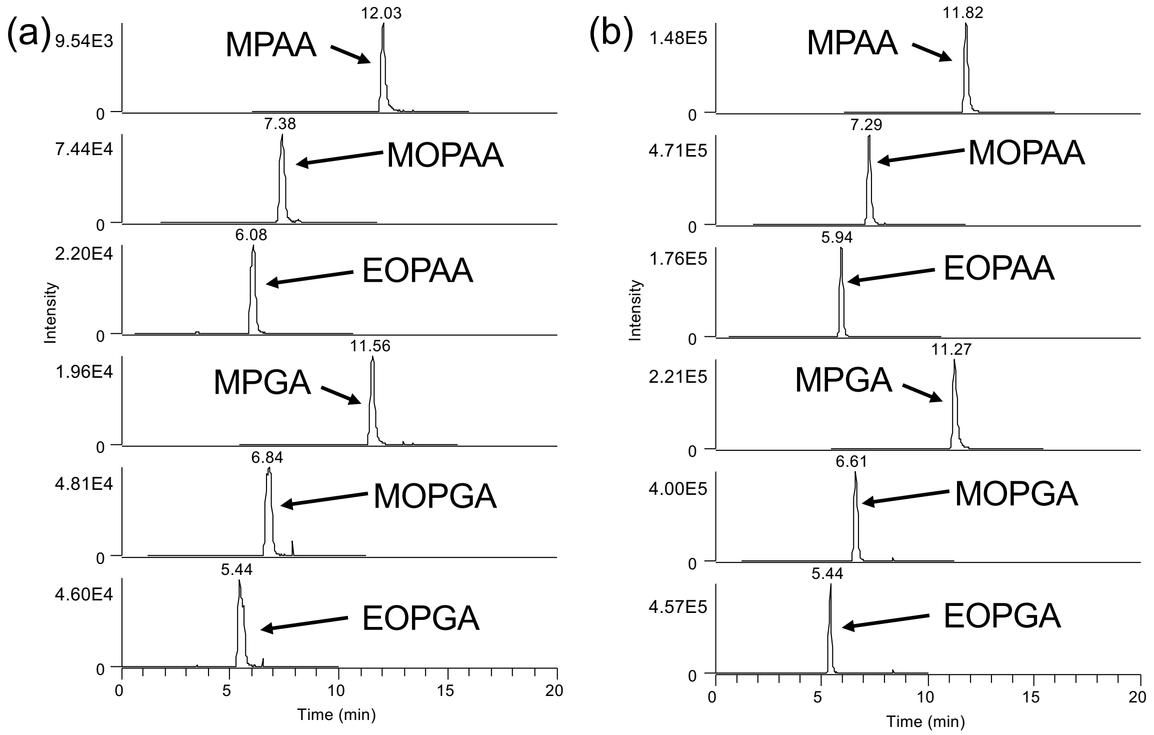

Fig. S1. EIC of urine sample analysis. (a) After dilution with water (10 times) and ultrafiltration, (b) after dilution with MeCN (10 times) and microfiltration.

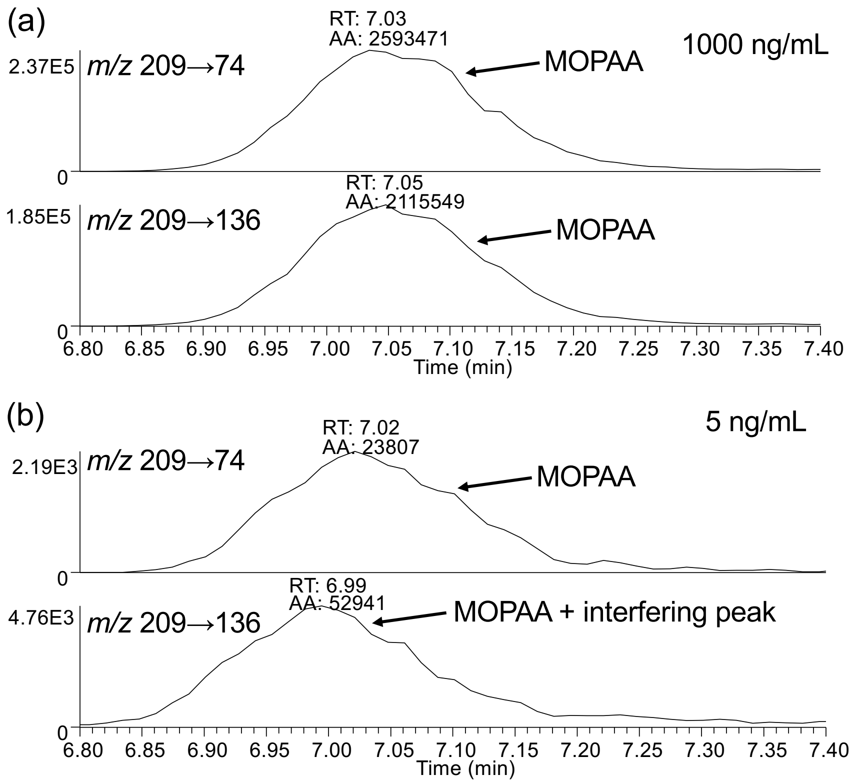

Fig. S2. EIC of urine sample after 10-fold dilution and microfiltration. (a) Urine with 1000 ng/mL of MOPAA, (b) urine with 5 ng/mL of MOPAA.

Table S3. Peak area errors calculated with constructed calibration curves for urine samples (%)

Concentration (ng/mL)	MOPAA	EOPAA	MPAA	MOPGA	EOPGA	MPGA
1000	–7.7	–7.8	–7.3	–5.6	–6.3	–6.5
500	+20.9	+20.4	+16	+18.6	+19.5	+15.6
250	–10.1	–11.6	–5.9	–15.5	–14.8	–7.7
100	–1.8	+3.9	+4.7	+1.6	+2.8	+5.6
50	–1.4	+14.1	+14.3	+10.5	+8.7	+10.3
25	–	–25.9	–15.2	–23.7	–26.1	–15.7
10	–	+2.1	+5.0	+5.1	+7.3	–1.5
5	–	+4.7	–11.5	+9.0	+8.8	–

Three samples were prepared for each concentration (n = 3).

3. Validation of the developed methods

3-1. Matrix effects

Table S4. Matrix effects of urine samples (%, average ± standard deviation, n = 3)

Concentration (ng/mL)	MOPAA	EOPAA	MPAA	MOPGA	EOPGA	MPGA
1000	151 ± 12	74 ± 7.4	127 ± 7.7	71 ± 8.6	93 ± 12	134 ± 8.0
500	142 ± 20	87 ± 3.1	161 ± 6.7	92 ± 1.5	110 ± 2.8	159 ± 6.7
50	105 ± 8.9	72 ± 1.7	150 ± 6.2	79 ± 3.1	100 ± 4.4	150 ± 6.6

4. Computational details for the calculation of physicochemical parameters

4-1. Calculation of pK_a

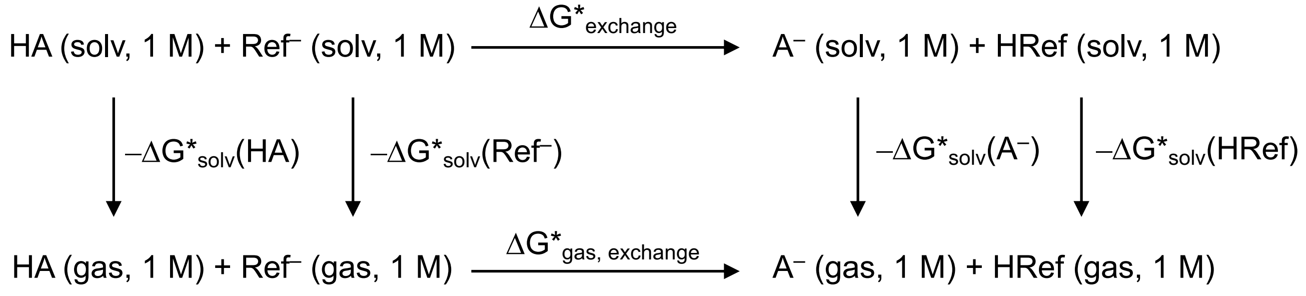

$$\Delta G^*_{exchange} = \Delta G^*_{gas,\ exchange} + \Delta G^*_{solv}(A^-) + \Delta G^*_{solv}(HRef) - \Delta G^*_{solv}(HA) - \Delta G^*_{solv}(Ref^-)$$

$$pK_a = \Delta G^*_{exchange}/RT\ ln\ (10) + pK_a\ (HRef)$$

Equation S1. Calculation of pK_a

4-2. Cartesian coordinates and energies

4-2-1. MOPAA

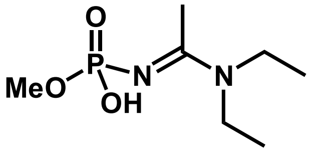

MOPAA

Gibbs Free Energy (M062X/6-311++G**)
 = -953.278365 (A. U.)

Gibbs Free Energy (SMD (H_2O)/M062X/6-311++G**)
 = -953.304426 (A. U.)

P	-1.59187900	-0.31161100	0.38102600
O	-1.43826100	0.66668600	1.50328700
N	-0.31718600	-1.18586800	-0.12103400
C	0.95626400	-1.03791000	-0.32657600
C	1.74050600	-2.33652300	-0.45642000
H	2.17158100	-2.43886200	-1.45884500
H	2.55709300	-2.40685900	0.26728700
H	1.04725800	-3.15795900	-0.28682200
N	1.65612800	0.12353900	-0.48273800
C	1.00646400	1.43524200	-0.66788800
H	1.46119800	1.88205700	-1.56201300
H	-0.04106100	1.26702500	-0.90897100
C	1.13131300	2.39576800	0.51694900
H	2.17480900	2.65131400	0.72757000
H	0.60633800	3.32677000	0.27641300
H	0.66409700	1.96932600	1.40724800
C	3.12953100	0.13035700	-0.55379900
H	3.47182300	-0.70748600	-1.16642400
H	3.41200000	1.03893200	-1.09367200
C	3.83650200	0.10740100	0.80819800
H	3.55836200	0.97079900	1.41702300
H	3.59144400	-0.79400400	1.37718000
H	4.92206000	0.12715800	0.65962800
O	-2.19336700	0.30031000	-1.00146900
C	-3.46365100	0.97568700	-0.97529600
H	-3.61733600	1.37715200	-1.97831600
H	-4.26353900	0.26890300	-0.73454900
H	-3.45396400	1.79139600	-0.24555400
O	-2.72441800	-1.43024300	0.75386000
H	-2.92977600	-1.36934000	1.69740200

MOPAA–H

Gibbs Free Energy (M062X/6-311++G**)
 = -952.737933 (A. U.)

Gibbs Free Energy (SMD (H_2O)/M062X/6-311++G**) = -952.853648 (A. U.)

P	-1.65062800	-0.37228500	0.53329400
O	-1.14155200	0.65530000	1.52511400
N	-0.35377100	-1.28931400	-0.11080000
C	0.88284700	-1.08745300	-0.36374300
C	1.72272000	-2.34872300	-0.60151700
H	2.17287100	-2.35970400	-1.60321200
H	2.53403500	-2.46280800	0.12725200

H	1.04747800	-3.19753500	-0.50447600
N	1.58597600	0.11547000	-0.50101400
C	0.92941300	1.40224300	-0.76410600
H	1.34926800	1.79023400	-1.70945500
H	-0.12933000	1.22026200	-0.93471600
C	1.09123300	2.45317300	0.33839800
H	2.14369600	2.71166500	0.51588700
H	0.57277300	3.37230500	0.03602900
H	0.62190900	2.08241300	1.25339500
C	3.04664600	0.14792100	-0.48203500
H	3.45974500	-0.66915000	-1.08473000
H	3.35489000	1.07522300	-0.98001600
C	3.67162800	0.10593200	0.92318700
H	3.32225300	0.94394500	1.53200200
H	3.40212000	-0.81582400	1.44817900
H	4.76719300	0.15305000	0.85488800
O	-2.11323300	0.43220800	-0.89634500
C	-3.47218700	0.82141800	-1.00099800
H	-3.63595300	1.17433300	-2.02751400
H	-4.13846000	-0.02343700	-0.78812300
H	-3.71317700	1.64023400	-0.30563200
O	-2.77410500	-1.31264400	0.89103300

4-2-2. EOPAA

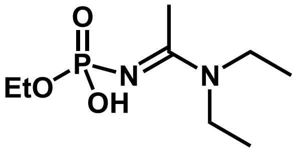

EOPAA

Gibbs Free Energy (M062X/6-311++G**)
= -992.560324 (A. U.)

Gibbs Free Energy (SMD (H_2O)/M062X/6-311++G**)
= -992.587481 (A. U.)

P	1.20951900	0.69767000	0.56030900
O	0.99965400	-0.00242300	1.86672800
N	-0.06641100	1.26078000	-0.27713100
C	-1.28245000	0.90975800	-0.56539000
C	-2.16426300	2.02949900	-1.10126900
H	-2.47244200	1.83000800	-2.13393500
H	-3.06912600	2.17205800	-0.50419600
H	-1.57987100	2.94720000	-1.08007600
N	-1.84277700	-0.33314600	-0.48948600
C	-1.05201500	-1.55926900	-0.27187300
H	-1.34135700	-2.25953500	-1.06690700

H	-0.00309500	-1.32318900	-0.43832100
C	-1.23697300	-2.21777500	1.09711100
H	-2.26953200	-2.54460100	1.25727000
H	-0.59787800	-3.10580500	1.15389500
H	-0.93263800	-1.53566200	1.89385300
C	-3.28761200	-0.54034700	-0.70159000
H	-3.62844600	0.07426000	-1.53864000
H	-3.41108400	-1.58074600	-1.01647100
C	-4.16036900	-0.27567600	0.53276500
H	-3.87947200	-0.92340300	1.36646300
H	-4.07727300	0.76034400	0.87352300
H	-5.21142900	-0.46654900	0.28826600
O	2.05111200	-0.13357500	-0.55456000
C	3.37794400	-0.61871900	-0.23625600
H	4.02940800	0.24319300	-0.05458700
H	3.32788100	-1.21961900	0.67883300
C	3.87280400	-1.44124200	-1.41228000
H	4.88282100	-1.81248800	-1.20671800
H	3.21916500	-2.30072000	-1.59073400
H	3.90470600	-0.83521400	-2.32277600
O	2.16091700	2.01154700	0.76803000
H	2.23534500	2.20022700	1.71404300

EOPAA–H

Gibbs Free Energy (M062X/6-311++G**)
= -992.019645 (A. U.)

Gibbs Free Energy (SMD (H_2O)/M062X/6-311++G**) = -
992.134561 (A. U.)

P	1.23896200	0.73478600	0.76375000
O	0.66096400	-0.09067700	1.89573700
N	-0.00880800	1.34290700	-0.24217100
C	-1.17776500	0.96379800	-0.59408300
C	-2.05731100	2.04432400	-1.23550500
H	-2.35325500	1.77872700	-2.25924500
H	-2.97385200	2.23975900	-0.66621900
H	-1.46383200	2.95700900	-1.26106700
N	-1.76880500	-0.30349400	-0.52424700
C	-0.99369900	-1.54269800	-0.38530100
H	-1.24093500	-2.17633500	-1.25586200
H	0.06332600	-1.29769800	-0.45799800
C	-1.24386000	-2.32621900	0.90686000
H	-2.29054000	-2.64365200	1.00522700
H	-0.62396800	-3.23213900	0.90209500
H	-0.94193600	-1.71127500	1.75862800

C	-3.21048000	-0.47918700	-0.68467400
H	-3.58483000	0.13104100	-1.51473100
H	-3.37724800	-1.52250400	-0.97875300
C	-4.03779700	-0.17906800	0.57717200
H	-3.72519100	-0.81433400	1.41004600
H	-3.91307400	0.86133400	0.89324800
H	-5.10532300	-0.35229000	0.38356000
O	1.97111500	-0.31916600	-0.35968200
C	3.37508200	-0.51468900	-0.26836100
H	3.86980100	0.45186500	-0.11143100
H	3.61282500	-1.15392300	0.59782700
C	3.86284800	-1.17776900	-1.55243100
H	4.94579700	-1.35544700	-1.50692800
H	3.36139500	-2.13997800	-1.71040800
H	3.65061500	-0.53865700	-2.41689300
O	2.22379500	1.84607900	1.02838400

4-2-3. MPAA

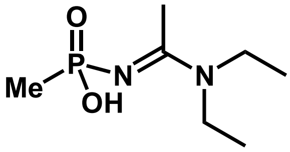

MPAA

Gibbs Free Energy (M062X/6-311++G**)
= -878.050002 (A. U.)

Gibbs Free Energy (SMD (H_2O)/M062X/6-311++G**)
= -878.075696 (A. U.)

P	1.96238900	0.02853200	0.03486100
O	2.20748400	0.98717600	1.16679100
N	0.70150100	-1.02107800	0.08170400
C	-0.59101500	-1.05549400	0.20825700
C	-1.20795200	-2.44409500	0.09401500
H	-1.65988100	-2.75120400	1.04433000
H	-1.98061700	-2.50192100	-0.67710700
H	-0.40955100	-3.14054000	-0.15383900
N	-1.44893500	-0.02369700	0.46084900
C	-0.98775300	1.32986100	0.82280700
H	-1.55586900	1.62814800	1.71302800
H	0.05658700	1.27542500	1.12866900
C	-1.16240600	2.38121900	-0.27792300
H	-2.21543600	2.57162900	-0.50876700
H	-0.72238200	3.32629900	0.05883200
H	-0.65147100	2.07268400	-1.19416300
C	-2.91151800	-0.21585800	0.44373200
H	-3.16190000	-1.16917900	0.91533100
H	-3.33580300	0.56203300	1.08540200
C	-3.55748300	-0.13553200	-0.94655200
H	-3.38570900	0.83753200	-1.41259700
H	-3.16563600	-0.90196700	-1.62141600
H	-4.63941400	-0.28589100	-0.85832700
C	3.38447600	-1.05256000	-0.28640300
H	4.28017600	-0.43968000	-0.41788100
H	3.52817000	-1.71377400	0.57210800
H	3.21150600	-1.65461700	-1.18127500
O	1.75143900	0.83795500	-1.39729600
H	2.13397800	1.72417000	-1.31666400

MPAA–H

Gibbs Free Energy (M062X/6-311++G**)
= -877.509965 (A. U.)

Gibbs Free Energy (SMD (H_2O)/M062X/6-311++G**) = -877.622982 (A. U.)

P	1.91431400	0.12947600	-0.10371900
O	2.21775400	0.83851900	1.21258200
N	0.71885000	-1.10446100	0.16568200
C	-0.55071500	-1.10183500	0.31026900
C	-1.23332600	-2.47406300	0.33845800
H	-1.78310100	-2.63943200	1.27496400
H	-1.94001200	-2.61589000	-0.48842900
H	-0.44608900	-3.22211600	0.25150800
N	-1.41719800	-0.01464900	0.49391400
C	-0.94643900	1.30720800	0.93108600
H	-1.54963100	1.58632000	1.81230200
H	0.08883700	1.21093600	1.26811500
C	-1.03186400	2.41533300	-0.12484600
H	-2.06344100	2.59515400	-0.45724500
H	-0.65432200	3.35032400	0.30851800
H	-0.39332500	2.15523700	-0.97496700
C	-2.85328800	-0.14297400	0.27093900
H	-3.23588500	-1.06087400	0.73423200
H	-3.33659300	0.68667800	0.80122200
C	-3.28625600	-0.10868600	-1.20604700
H	-2.97172200	0.82231000	-1.68484000
H	-2.83809700	-0.93428200	-1.76806100
H	-4.37902600	-0.19407600	-1.28567000
C	3.34695000	-0.96149600	-0.51092200
H	4.21319900	-0.32198500	-0.71203500
H	3.57566600	-1.62209600	0.33137600

H	3.13076600	-1.56256900	-1.39995000
O	1.54301300	0.91861500	-1.35333900

4-2-4. MOPGA

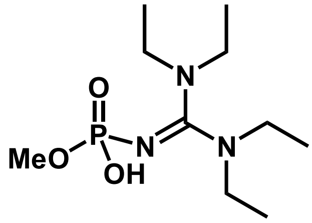

MOPGA
Gibbs Free Energy (M062X/6-311++G**)
 = -1126.439834 (A. U.)
Gibbs Free Energy (SMD (H_2O)/M062X/6-311++G**)
 = -1126.464965 (A. U.)

P	-1.82200800	-0.82097400	0.37408700
O	-2.29653900	0.16115500	1.40082100
H	-2.45849300	-2.31095000	1.79037300
O	-2.11701800	-2.34936300	0.88579300
O	-2.62912500	-0.77347400	-1.03797500
C	-4.06135800	-0.89195400	-1.02630300
H	-4.35742200	-1.89412400	-0.69975100
H	-4.39138000	-0.73105900	-2.05406200
H	-4.50858100	-0.13870000	-0.37006700
N	-0.26884600	-0.87034500	-0.11085000
C	0.66993500	0.03706400	-0.15027400
N	0.49974400	1.39804200	-0.30274300
N	1.98643400	-0.39117200	-0.03900800
C	-0.59227300	1.95392400	-1.12104000
C	1.34998500	2.35981600	0.42177700
C	2.97961000	0.06754400	-1.02770700
C	2.22388300	-1.71114300	0.56680000
H	-0.12477100	2.53287800	-1.93287300
H	-1.11323500	1.11672400	-1.58724800
C	-1.59509900	2.84471800	-0.38015300
H	2.40667000	2.11140800	0.30324000
H	1.20506100	3.33235600	-0.05931100
C	1.01860400	2.45113600	1.91575300
H	3.92944100	0.26974500	-0.51867800
H	2.62719500	1.01362100	-1.43851300
C	3.20249400	-0.91330400	-2.18772600
H	1.41032800	-1.88605100	1.27063000
H	2.14520700	-2.50585200	-0.18690100
C	3.56946700	-1.79105300	1.29105000
H	-1.11956400	3.74397300	0.02581300
H	-2.36269400	3.17652200	-1.08861400
H	-2.07875400	2.29720800	0.43191000
H	-0.02691700	2.72159300	2.08013900
H	1.19655400	1.49049000	2.40870100
H	1.65748300	3.20270500	2.39429600
H	2.26015700	-1.13620700	-2.69835100
H	3.88882300	-0.46446900	-2.91417100
H	3.64298100	-1.85848900	-1.85772600
H	3.64804300	-2.75777100	1.79920600
H	4.42402200	-1.71144000	0.61105800
H	3.65860200	-1.00220300	2.04562800

MOPGA–H
Gibbs Free Energy (M062X/6-311++G**)
 = -1125.901087 (A. U.)
Gibbs Free Energy (SMD (H_2O)/M062X/6-311++G**)
 = -1126.009078 (A. U.)

P	1.85315200	-0.83692100	-0.61675000
O	2.06669300	0.40135800	-1.46459200
O	2.26642900	-2.20137800	-1.11084200
O	2.59671100	-0.59866900	0.89248300
C	3.90936000	-1.10934400	1.05346400
H	3.96359700	-2.15842400	0.73843100
H	4.64259200	-0.53141300	0.47019000
N	0.22885100	-0.93705100	-0.06348400
C	-0.67010700	-0.04418600	0.09829800
N	-0.53186500	1.32875200	0.34534400
N	-2.04599700	-0.43712000	0.05827700
C	0.56701900	1.85427700	1.16156500
C	-1.39064700	2.29640400	-0.33723100
C	-2.83827600	-0.14732700	1.25960900
C	-2.34237200	-1.71946500	-0.58432600
H	0.11768600	2.36551100	2.03399500
H	1.13517900	1.00108200	1.53379900
C	1.53326000	2.81630700	0.45935800
H	-2.44323900	2.00914000	-0.24151300
H	-1.27636600	3.25447100	0.18544400
C	-1.06267900	2.46983300	-1.82769700
H	-3.89253800	-0.33810800	1.02303400
H	-2.75701800	0.91928400	1.48725300
C	-2.43385900	-0.94745900	2.51054400
H	-1.48187800	-1.96906900	-1.20616200
H	-2.41503900	-2.53184700	0.15587500

C	-3.62205600	-1.66661400	-1.42721600
H	1.03033700	3.73116100	0.11997300
H	2.31262700	3.11655200	1.17198900
H	1.99899500	2.30670100	-0.38928800
H	-0.01853000	2.75359600	-1.97654700
H	-1.21894700	1.52735400	-2.36131600
H	-1.71544200	3.23364600	-2.27323900
H	-1.39173500	-0.74720600	2.77740500
H	-3.06993100	-0.66500600	3.35951200
H	-2.53501700	-2.02656300	2.35656600
H	-3.79812300	-2.63966400	-1.90277500
H	-4.51061600	-1.42763500	-0.82952700
H	-3.53344000	-0.91041100	-2.21481200
H	4.16611700	-1.02645200	2.11741400

4-2-5. EOPGA

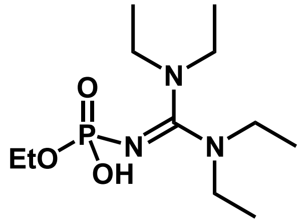

EOPGA

Gibbs Free Energy (M062X/6-311++G**)
 = -1165.7215 (A. U.)

Gibbs Free Energy (SMD (H_2O)/M062X/6-311++G**)
 = -1165.746636 (A. U.)

P	-1.54012000	-0.63913600	0.76652500
O	-1.82691200	0.45066100	1.75367600
O	-1.83258200	-2.09998800	1.44991900
O	-2.52043700	-0.66520700	-0.52995200
H	-2.05002100	-1.96446800	2.38301300
C	-3.95321700	-0.70829000	-0.33836400
H	-4.21149200	-1.64894500	0.16177300
H	-4.25352200	0.12512600	0.30680400
C	-4.61449500	-0.61830500	-1.70209400
H	-4.29738900	-1.44751200	-2.34179100
H	-5.70350200	-0.66218500	-1.59081400
H	-4.35634000	0.32164600	-2.19971100
N	-0.06654600	-0.81844900	0.09579600
C	0.90634900	0.02217600	-0.13117600
N	0.79280900	1.37763500	-0.36502300
N	2.20184100	-0.48010100	-0.15443000
C	-0.36692100	1.94604000	-1.07408800
C	1.78632000	2.32967100	0.16357100
C	3.07472900	-0.16369300	-1.30000900
C	2.44358900	-1.76586900	0.51937700
H	0.01765500	2.43323000	-1.98387700
H	-0.99309600	1.11452100	-1.39993600
C	-1.20680300	2.95242600	-0.28051800
H	2.80139200	2.00445900	-0.07331900
H	1.63187600	3.27354200	-0.36888200
C	1.66763600	2.54734100	1.67640100
H	4.09587600	0.00759200	-0.93934100
H	2.72615600	0.77308900	-1.73485500
C	3.07745300	-1.23774200	-2.39727200
H	1.72515100	-1.83385300	1.33617500
H	2.21738900	-2.60627300	-0.15028500
C	3.86861500	-1.88390500	1.06425200
H	-0.62873700	3.84268800	-0.01062700
H	-2.04161900	3.28701600	-0.90698400
H	-1.60850600	2.49585500	0.62683600
H	0.67185600	2.89928400	1.95549900
H	1.85525800	1.61345700	2.21506600
H	2.40837800	3.28522600	2.00636800
H	2.06237900	-1.43006600	-2.75929700
H	3.68325100	-0.89098200	-3.24168300
H	3.50172400	-2.18556000	-2.05378900
H	3.96017400	-2.81394800	1.63486600
H	4.62542800	-1.91253000	0.27340800
H	4.10527700	-1.04979900	1.73349700

EOPGA–H

Gibbs Free Energy (M062X/6-311++G**)
 = -1165.182892 (A. U.)

Gibbs Free Energy (SMD (H_2O)/M062X/6-311++G**)
 = -1165.292554 (A. U.)

P	-1.55614900	-0.48030300	1.08516100
O	-1.53693900	0.88398800	1.74450200
O	-1.99860500	-1.70801200	1.84270900
O	-2.49986100	-0.38865400	-0.32641500
C	-3.85170100	-0.81921800	-0.24913100
H	-3.90500900	-1.76420000	0.30569000
H	-4.45427900	-0.07741500	0.30021100
C	-4.39561300	-0.98362500	-1.66437300
H	-3.81661000	-1.73595300	-2.21172500
H	-5.44604200	-1.30309600	-1.63815100
H	-4.33590500	-0.03930100	-2.21825200

N	-0.04864600	-0.83036800	0.33724700
C	0.89140300	-0.06996500	-0.07499300
N	0.83823900	1.26430100	-0.50151500
N	2.21751700	-0.60074900	-0.16398200
C	-0.31767600	1.78803200	-1.23591700
C	1.86938200	2.21760700	-0.09242000
C	2.84662600	-0.55781200	-1.48960100
C	2.49274100	-1.81153900	0.61282400
H	0.04122600	2.11851200	-2.22914100
H	-1.00820300	0.95879900	-1.39300700
C	-1.07957000	2.94034100	-0.56977000
H	2.86693800	1.80445900	-0.27575400
H	1.76914300	3.09923000	-0.73817600
C	1.77469400	2.63444600	1.38288500
H	3.90298700	-0.82949200	-1.37086400
H	2.82804700	0.47237200	-1.85621500
C	2.19262100	-1.46581400	-2.54614400
H	1.71282500	-1.88596200	1.37154000
H	2.38822900	-2.71691200	-0.00571500
C	3.88060300	-1.78302200	1.26385100
H	-0.45184600	3.83220800	-0.44530500
H	-1.92464200	3.22231700	-1.21143100
H	-1.45981900	2.60969000	0.40113400
H	0.79281000	3.05084600	1.61818600
H	1.91892800	1.76495100	2.03141900
H	2.55122900	3.37590600	1.61791300
H	1.14527000	-1.18851600	-2.69940100
H	2.72060500	-1.36943100	-3.50375600
H	2.21678200	-2.51977500	-2.25125700
H	4.04020800	-2.69883200	1.84677300
H	4.69040900	-1.71768800	0.52640500
H	3.97136700	-0.92562300	1.93956700

4-2-6. MPGA

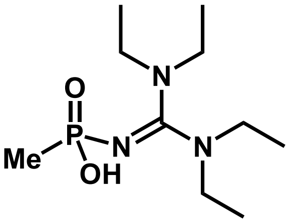

MPGA

Gibbs Free Energy (M062X/6-311++G**)
= -1051.210675 (A. U.)

Gibbs Free Energy (SMD (H_2O)/M062X/6-311++G**)
= -1051.23349 (A. U.)

P	-2.00953400	-1.15003100	-0.07564000
O	-2.98941000	-0.47797900	-0.99498800
N	-0.38722900	-0.96616200	-0.28991100
C	0.43003600	0.04629900	-0.18156700
N	0.11201400	1.38822000	-0.26073400
C	-0.92345300	1.88587400	-1.18456800
H	-0.42043300	2.53901000	-1.91497700
H	-1.33332000	1.03258200	-1.72342600
C	-2.07359700	2.65718700	-0.53403900
H	-1.73188400	3.56318800	-0.02226600
H	-2.77979100	2.96027100	-1.31372500
H	-2.61566000	2.02783900	0.17432100
C	0.80034600	2.38888900	0.57383200
H	1.87780100	2.21647600	0.57769100
H	0.64217900	3.36282400	0.09897400
C	0.29702100	2.41594800	2.02204200
H	-0.77079900	2.64262100	2.07348300
H	0.45857000	1.44694400	2.50388400
H	0.84186600	3.17728800	2.59269100
O	-2.26310500	-0.69557600	1.50009200
H	-3.14659000	-0.30764400	1.58015100
N	1.77671100	-0.23041700	0.04112500
C	2.78293900	0.37905000	-0.84854700
H	3.67246300	0.63771500	-0.26222100
H	2.36858500	1.31244400	-1.23033200
C	3.17737300	-0.50260400	-2.04216800
H	2.29787300	-0.77778800	-2.63303100
H	3.86641500	0.05043200	-2.68995000
H	3.68162100	-1.42278900	-1.73292900
C	2.11437300	-1.54486500	0.60741100
H	1.28168000	-1.83252700	1.24968100
H	2.17047600	-2.30964600	-0.17932100
C	3.41143700	-1.51932000	1.41922100
H	3.55581300	-2.49290200	1.89914500
H	4.29551300	-1.32763800	0.80236800
H	3.36991700	-0.75643400	2.20398600
C	-2.21038200	-2.95473700	-0.04197900
H	-1.56929800	-3.40079200	0.72199100
H	-3.25638900	-3.19406100	0.16696700
H	-1.94050500	-3.36032600	-1.02056900

MPGA–H

Gibbs Free Energy (M062X/6-311++G**)
= -1050.672373 (A. U.)

Gibbs Free Energy (SMD (H_2O)/M062X/6-311++G**)
= -1050.781339 (A. U.)

P	-2.08069400	-1.09239900	0.09121000
O	-2.70487300	-0.93627800	-1.29052100
N	-0.35039900	-0.96577700	-0.06070500
C	0.42565800	0.04442500	-0.11355800
N	0.12454400	1.39312700	-0.35004200
C	-1.03731100	1.79443200	-1.15351200
H	-0.66628900	2.43888100	-1.97110800
H	-1.46343200	0.89343100	-1.60203600
C	-2.14963700	2.52693900	-0.39199600
H	-1.80419200	3.47883600	0.03344100
H	-2.96609200	2.75167500	-1.08967500
H	-2.54075900	1.88166800	0.40102000
C	0.84103700	2.45049400	0.36327400
H	1.92371700	2.34337900	0.23184100
H	0.55939400	3.39882600	-0.11017400
C	0.53229400	2.51070700	1.86671800
H	-0.53849400	2.63366200	2.04754100
H	0.84546100	1.58231100	2.35400000
H	1.07410400	3.34509900	2.33395500
O	-2.55061600	-0.24784000	1.26897400
N	1.84096600	-0.15222100	0.07402000
C	2.66640200	0.17019300	-1.09909400
H	3.70639600	0.30178200	-0.77256800
H	2.33139700	1.13868600	-1.47936500
C	2.60791500	-0.85187800	-2.24866600
H	1.57442200	-1.01401000	-2.56979800
H	3.18557800	-0.48195600	-3.10544400
H	3.02504300	-1.82256000	-1.95971700
C	2.20205500	-1.38568700	0.77494900
H	1.44241800	-1.54031600	1.54320700
H	2.13842800	-2.26887500	0.12012800
C	3.58967600	-1.31200000	1.42156700
H	3.76530200	-2.21298000	2.02190300
H	4.39846500	-1.25138300	0.68372500
H	3.66714400	-0.43935900	2.08030400
C	-2.18598500	-2.87171800	0.57356700
H	-1.66381600	-3.04800500	1.51973800
H	-3.24418400	-3.12535200	0.69801000
H	-1.75410000	-3.50586500	-0.20710200

4-2-7. CH_3COOH (HRef for degradation products of Novichok agents)

CH_3COOH
pK_a (H_2O) = 4.76 (Evans's pK_a Table)
Gibbs Free Energy (M062X/6-311++G**)
= -229.028596 (A. U.)
Gibbs Free Energy (SMD (H_2O)/M062X/6-311++G**) = -229.040363 (A. U.)

C	-1.39693800	-0.12129800	0.00001100
H	-1.67707800	-0.70495200	-0.88222100
H	-1.67709500	-0.70625100	0.88135400
H	-1.92406500	0.83200200	0.00063500
C	0.08839700	0.12494900	0.00004100
O	0.63284300	1.20849300	-0.00000400
O	0.79152500	-1.03864400	0.00000600
H	1.73454100	-0.80149000	-0.00009600

CH_3COOH–H
Gibbs Free Energy (M062X/6-311++G**)
= -228.47821 (A. U.)
Gibbs Free Energy (SMD (H_2O)/M062X/6-311++G**) = -228.593273 (A. U.)

C	-1.35469800	-0.02130600	0.00414900
H	-1.74242600	-0.98724700	0.34697800
H	-1.75647700	0.78902600	0.62420400
H	-1.70651300	0.14588700	-1.02350500
C	0.20896400	0.00050300	0.00995700
O	0.73340200	1.14858500	-0.00200800
O	0.77657600	-1.12644100	-0.00203100

4-2-8. MPA

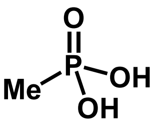

MPA
Gibbs Free Energy (M062X/6-311++G**)
= -608.139028 (A. U.)
Gibbs Free Energy (SMD (H_2O)/M062X/6-311++G**)
= -608.177219 (A. U.)

P	-0.09987400	0.14460100	0.00057000
O	-0.68621100	1.49867400	0.00579200
O	-0.57051700	-0.72610300	1.29930800

C	1.71686500	0.06846000	-0.00096700
H	2.08800300	0.58164600	0.88975900
H	2.09527300	-0.95921200	-0.00470300
H	2.08663600	0.58754500	-0.88883200
O	-0.57233700	-0.71655900	-1.30381800
H	-0.22303600	-1.61469300	-1.38357500
H	-0.21743700	-1.62316800	1.37433900

MPA–H

Gibbs Free Energy (M062X/6-311++G**)
= -607.62283 (A. U.)

Gibbs Free Energy (SMD (H_2O)/M062X/6-311++G**) = -607.738438 (A. U.)

P	-0.09552300	-0.15040500	0.09080200
O	-0.15116900	-1.57772800	-0.40162600
O	-0.80602100	0.85342800	-1.08338300
C	1.65819100	0.42882000	-0.01992000
H	2.04339200	0.27096800	-1.03253800
H	1.73416300	1.48975000	0.24020200
H	2.26345100	-0.15438100	0.68235500
O	-0.68870300	0.30419100	1.41605500
H	-1.39016000	1.43769400	-0.58089500

4-2-9. EMPA

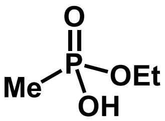

EMPA

Gibbs Free Energy (M062X/6-311++G**)
= -686.684063 (A. U.)

Gibbs Free Energy (SMD (H_2O)/M062X/6-311++G**) = -686.714202 (A. U.)

P	0.89200600	-0.17041900	-0.09238700
O	1.70763500	-1.17590600	-0.80469900
O	-0.68886200	-0.31937400	-0.42452200
C	-1.74491900	0.20883400	0.40301200
H	-1.70207800	-0.27443800	1.38573600
C	-3.06943500	-0.07353900	-0.28289500
H	-3.20420300	-1.14966600	-0.42261300
H	-3.89417800	0.30594900	0.32982800
H	-3.11039000	0.41068800	-1.26274800
C	1.30775100	1.56318900	-0.45755300
H	1.20821100	1.71526700	-1.53527400
H	0.65274200	2.27111600	0.06052500
H	2.34544100	1.75067700	-0.17004300
O	1.00621300	-0.30918900	1.53510600
H	0.76927200	0.48023500	2.04076000
H	-1.60517400	1.29130200	0.53717000

EMPA–H

Gibbs Free Energy (M062X/6-311++G**)
= -686.16364 (A. U.)

Gibbs Free Energy (SMD (H_2O)/M062X/6-311++G**) = -686.274559 (A. U.)

P	0.91573400	-0.21128800	0.06460500
O	1.64516700	-1.21429600	-0.79552900
O	-0.67726200	-0.16193200	-0.53208700
C	-1.70651600	0.36448000	0.28871900
H	-1.54798400	0.05859900	1.33015000
C	-3.05336700	-0.13274800	-0.22807500
H	-3.09590200	-1.22646600	-0.18334100
H	-3.87525300	0.27587000	0.37577200
H	-3.20518500	0.16847600	-1.27120200
C	1.48877800	1.48875600	-0.39790500
H	1.45157700	1.61718200	-1.48442200
H	0.87444700	2.25731600	0.08504700
H	2.52381800	1.60996600	-0.06094500
O	0.83884500	-0.29679900	1.57748900
H	-1.68888300	1.46965400	0.26445000

4-2-10. IMPA

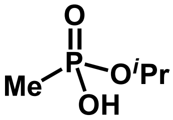

IMPA

Gibbs Free Energy (M062X/6-311++G**)
= -725.979407 (A. U.)

Gibbs Free Energy (SMD (H_2O)/M062X/6-311++G**) = -725.999311 (A. U.)

P	1.06073600	-0.11142900	0.06845300
O	1.21912100	-1.43276900	0.74162800
O	-0.38225300	0.23022100	-0.56114700
C	-1.61338000	0.02937200	0.20996600

H	-1.35361000	0.02904100	1.27522900
C	-2.21567700	-1.31700800	-0.17154200
H	-1.51788200	-2.12752800	0.05333600
H	-3.14070400	-1.48690200	0.39048100
H	-2.45197100	-1.33906800	-1.24074700
C	-2.51529400	1.21698400	-0.09476300
H	-2.03058700	2.15498400	0.18979700
H	-2.74761100	1.25530400	-1.16421800
H	-3.45521100	1.12730500	0.46020600
C	2.13729200	0.23165700	-1.34067800
H	1.92577900	-0.48596600	-2.13673300
H	1.97181000	1.24699300	-1.70754600
H	3.17646100	0.12015600	-1.02208500
O	1.32649700	1.13051500	1.10743100
H	1.64792200	0.78734600	1.95429000

IMPA–H
Gibbs Free Energy (M062X/6-311++G**)
= -725.446948 (A. U.)
Gibbs Free Energy (SMD (H_2O)/M062X/6-311++G**) = -725.555401 (A. U.)

P	1.11253800	0.11754100	-0.15119700
O	1.34271500	1.57047900	0.21720000
O	-0.37760500	-0.39132900	0.50640000
C	-1.56545400	-0.05649600	-0.20758900
H	-1.32832700	-0.00339400	-1.28009100
C	-2.10251800	1.30305800	0.25709900
H	-1.32737400	2.06441200	0.13473000
H	-2.99227000	1.59259500	-0.31982600
H	-2.37469000	1.25975400	1.31969900
C	-2.57315900	-1.18598900	0.01521000
H	-2.16526000	-2.13381000	-0.35078800
H	-2.78928500	-1.29561500	1.08563600
H	-3.51581400	-0.98208200	-0.51006200
C	2.17121900	-0.95983000	0.90498800
H	2.00456300	-0.73606500	1.96326500
H	1.95899500	-2.01576800	0.71243400
H	3.21759700	-0.75243800	0.65844000
O	1.16529700	-0.34979500	-1.59406600

4-2-11. ^{i}BuMPA

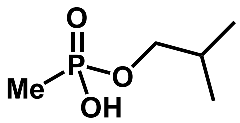

^{i}BuMPA
Gibbs Free Energy (M062X/6-311++G**)
= -765.24055 (A. U.)
Gibbs Free Energy (SMD (H_2O)/M062X/6-311++G**) = -765.269633 (A. U.)

P	1.64208800	-0.17005000	-0.02020700
O	2.41317400	-1.38324200	-0.36353400
O	0.06627900	-0.33044500	-0.36607600
C	-0.98099000	0.48196400	0.19824200
H	-0.98027100	0.36256800	1.28987900
C	-2.32449200	0.03751400	-0.38076300
H	-2.24875600	0.13195500	-1.47276600
C	2.17723400	1.35104000	-0.86312000
H	2.11928700	1.18032400	-1.94111800
H	1.55420800	2.21443200	-0.60831300
H	3.21574000	1.56094800	-0.59482700
O	1.70434100	0.17580200	1.57842200
H	1.45510100	1.07794000	1.82195000
H	-0.79931600	1.53931800	-0.04350900
C	-3.43282600	0.97907500	0.11484100
H	-3.23764300	2.02190800	-0.16152200
H	-4.39790800	0.69622000	-0.31727300
H	-3.53507400	0.93164200	1.20626400
C	-2.62646700	-1.42760900	-0.03892500
H	-1.82886200	-2.08712200	-0.39019800
H	-2.72689600	-1.56253500	1.04563000
H	-3.56602500	-1.74567100	-0.50324400

----

^{i}BuMPA–H
Gibbs Free Energy (M062X/6-311++G**)
= -764.721624 (A. U.)
Gibbs Free Energy (SMD (H_2O)/M062X/6-311++G**) = -764.829766 (A. U.)

P	-1.62910600	0.17694100	0.12039500
O	-2.30442000	1.35820800	-0.53285000
O	-0.10305800	0.05166900	-0.62353400
C	0.94883800	-0.63294400	0.03226600
H	0.82917100	-0.55036500	1.12159100
C	2.29966300	-0.04765500	-0.40428900
H	2.34148500	-0.11450100	-1.50202500

C	-2.42063300	-1.37641700	-0.50264000
H	-2.47695400	-1.35945900	-1.59574000
H	-1.86027500	-2.26001500	-0.17652100
H	-3.43547200	-1.43834800	-0.09550000
O	-1.42351700	0.04692200	1.61773100
H	0.92308700	-1.70731300	-0.22833400
C	3.45555500	-0.87747200	0.17468700
H	3.39119800	-1.92888200	-0.13330200
H	4.42778600	-0.48976000	-0.15516400
H	3.44366400	-0.85167700	1.27247400
C	2.41412500	1.43134200	-0.01310300
H	1.56380500	1.99269900	-0.40923400
H	2.40802000	1.54394100	1.07914100
H	3.34373600	1.87404700	-0.39561100

4-2-12. CHMPA

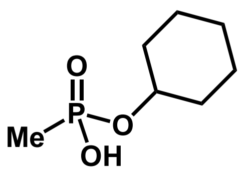

CHMPA
Gibbs Free Energy (M062X/6-311++G**)
= -842.620243 (A. U.)
Gibbs Free Energy (SMD (H_2O)/M062X/6-311++G**) = -842.650227 (A. U.)

P	2.10577900	-0.06553500	-0.10918700
O	3.12839200	-0.36100900	-1.13482700
O	0.63036100	0.11978500	-0.75016700
C	2.40793900	1.43606300	0.87387700
H	2.48428400	2.28274900	0.18700800
H	1.60609100	1.63490500	1.59222600
H	3.35517000	1.32767800	1.40815300
O	1.97405000	-1.27454900	0.98827400
H	1.46935100	-1.06123500	1.78528100
C	-0.62004800	0.08152800	-0.01765400
C	-1.45845500	1.28714900	-0.44255200
C	-1.33603100	-1.24029800	-0.30174700
H	-0.41640300	0.15851700	1.06337700
C	-2.84768500	1.25846700	0.21815600
H	-1.55924800	1.25972100	-1.53515200
H	-0.92622300	2.21292100	-0.19504700
C	-2.72429400	-1.27232600	0.36098200
H	-1.43534600	-1.33951100	-1.39029200
H	-0.71628900	-2.07701300	0.03800000
C	-3.57844700	-0.06530200	-0.05805700
H	-3.44205100	2.10721400	-0.13927300
H	-2.73975600	1.39272600	1.30468500
H	-3.23175700	-2.20941500	0.10489900
H	-2.60989400	-1.27286900	1.45526500
H	-4.54151100	-0.08183800	0.46596700
H	-3.80341000	-0.13702800	-1.13156400

CHMPA–H
Gibbs Free Energy (M062X/6-311++G**)
= -842.100373 (A. U.)
Gibbs Free Energy (SMD (H_2O)/M062X/6-311++G**) = -842.209535 (A. U.)

P	2.10305800	-0.19960300	0.00270900
O	3.02250100	-0.58874200	-1.12884700
O	0.64552500	0.30162800	-0.72408500
C	2.67604800	1.41682800	0.70052700
H	2.82010400	2.14526900	-0.10366500
H	1.95401500	1.80836800	1.42598000
H	3.63166400	1.25674300	1.21118700
O	1.75157400	-1.12374500	1.15451400
C	-0.58020700	0.17124300	-0.01688500
C	-1.50059200	1.32925500	-0.42638800
C	-1.24106200	-1.18403200	-0.31090900
H	-0.39336400	0.22319400	1.06701400
C	-2.88570400	1.22180900	0.23448500
H	-1.60591400	1.31346200	-1.52089300
H	-1.01940400	2.28127100	-0.16982900
C	-2.62341600	-1.29984200	0.35300000
H	-1.33725700	-1.28677700	-1.40189600
H	-0.56632000	-1.97160100	0.03722400
C	-3.54603600	-0.13768900	-0.05098900
H	-3.53528700	2.04017900	-0.10684600
H	-2.77865200	1.34372900	1.32316000
H	-3.09005600	-2.26120700	0.09727800
H	-2.50053400	-1.29699300	1.44655500
H	-4.51063400	-0.21008300	0.47172100
H	-3.76522600	-0.21006800	-1.12731700

4-2-13. PMPA

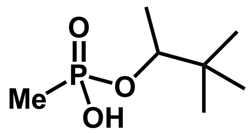

PMPA
Gibbs Free Energy (M062X/6-311++G**)
= -843.815638 (A. U.)
Gibbs Free Energy (SMD (H_2O)/M062X/6-311++G**) = -843.833795 (A. U.)

P	-1.85440700	0.04100300	0.06939300
O	-2.35140800	1.32121400	0.65145800
O	-0.34826200	0.02142000	-0.50059100
C	0.77272400	0.59474300	0.25491800
C	2.01486700	-0.30021700	-0.00684200
C	-2.76119400	-0.62911500	-1.34210200
H	-2.69305100	0.07060700	-2.17822800
H	-2.34773700	-1.59600500	-1.63741000
H	-3.80919600	-0.74983200	-1.05758700
O	-1.86795700	-1.16949900	1.17574800
H	-2.27999700	-0.86051700	1.99611200
H	0.53146100	0.51891700	1.32318800
C	3.20950600	0.25946400	0.79475200
H	2.96613200	0.36473400	1.85914100
H	4.06139100	-0.42473300	0.71775700
H	3.53698100	1.23499900	0.42263800
C	2.37228300	-0.34609000	-1.50555100
H	1.53067100	-0.71225500	-2.10082100
H	2.65960500	0.63852200	-1.88871000
H	3.22027500	-1.02110700	-1.66622300
C	1.71607100	-1.72979400	0.49043400
H	2.59403900	-2.36989900	0.34963900
H	1.46464200	-1.73502600	1.55749100
H	0.87834500	-2.17504200	-0.05189600
C	0.89214500	2.06772700	-0.12665300
H	1.10191900	2.18410300	-1.19375900
H	-0.04700700	2.57812800	0.09957800
H	1.69022400	2.55397000	0.44153700

PMPA–H
Gibbs Free Energy (M062X/6-311++G**)
= -843.286618 (A. U.)
Gibbs Free Energy (SMD (H_2O)/M062X/6-311++G**) = -843.390756 (A. U.)

P	1.88456200	-0.08799800	-0.21464800
O	2.23874900	1.25629500	-0.82277500
O	0.35843100	0.05756000	0.53404200
C	-0.71977000	0.58027900	-0.24147800
C	-1.98497500	-0.29586700	0.02176100
C	2.84179200	-0.35382300	1.33771100
H	2.76439600	0.51975100	1.99195000
H	2.47948500	-1.24272500	1.86337100
H	3.89120900	-0.50612500	1.06543600
O	1.89292500	-1.35835200	-1.04051000
H	-0.48517600	0.47944700	-1.31205700
C	-3.19328200	0.26575300	-0.75550700
H	-2.95705000	0.38620400	-1.82058800
H	-4.04650700	-0.42117500	-0.68024100
H	-3.51619700	1.23911200	-0.36951700
C	-2.31929900	-0.34672400	1.52541700
H	-1.46176100	-0.72158000	2.09127000
H	-2.57943700	0.64342100	1.91835900
H	-3.17540000	-1.01107400	1.70460700
C	-1.69345800	-1.72454400	-0.48064500
H	-2.53788600	-2.39175700	-0.25853900
H	-1.52697800	-1.73155300	-1.56420800
H	-0.78628300	-2.12070300	-0.01857200
C	-0.86510200	2.07666700	0.06399900
H	-1.11339800	2.24421400	1.11868800
H	0.09916000	2.54695400	-0.14577400
H	-1.63288200	2.55308200	-0.55807100

4-2-14. CH_3COOH (HRef for degradation products of conventional nerve agents)

CH_3COOH
pK_a (H_2O) = 4.76 (Evans's pK_a Table)
Gibbs Free Energy (M062X/6-311++G**)
= -229.019171 (A. U.)
Gibbs Free Energy (SMD (H_2O)/M062X/6-311++G**) = -229.039201 (A. U.)

C	-1.37054200	-0.16083300	-0.00001200
H	-1.61717100	-1.22612100	-0.00004800
H	-1.81654400	0.30755300	-0.88157600
H	-1.81665400	0.30751900	0.88151400
C	0.11981900	0.13558700	0.00009200
O	0.96226800	-0.93653900	-0.00001500
H	0.46708500	-1.76723400	-0.00003600

O	0.57368500	1.25275800	-0.00002700

CH_3COOH–H

Gibbs Free Energy (M062X/6-311++G**)
= -228.478409 (A. U.)

Gibbs Free Energy (SMD (H_2O)/M062X/6-311++G**) = -228.593406 (A. U.)

C	-1.35396400	-0.04874400	-0.00000100
H	-1.72733900	-1.07870800	-0.00005200
H	-1.73878700	0.47959800	-0.88222600
H	-1.73878300	0.47950600	0.88228200
C	0.20906300	0.00117500	0.00000100
O	0.80406200	-1.11135800	0.00000000
O	0.70522800	1.16198600	0.00000000
